# Editorial: Advances in smart and adaptive prosthetic and wearable technologies

**DOI:** 10.3389/frobt.2026.1934269

**Published:** 2026-08-11

**Authors:** Charikleia Angelidou, Lucas Ferrari Gerez, Kinsey Herrin, Cara Gonzalez Welker

**Affiliations:** 1 Department of Mechanical Engineering, University of Delaware, Newark, DE, United States; 2 James Watt School of Engineering, University of Glasgow, Glasgow, United Kingdom; 3 George W. Woodruff School of Mechanical Engineering, Georgia Institute of Technology, Atlanta, GA, United States; 4 Department of Mechanical Engineering, University of Colorado Boulder, Boulder, CO, United States

**Keywords:** adaptive control, gait rehabilitation, human-machine interface, hybrid actuation, lower-limb prostheses, machine learning, wearable robotics

## 1 Introduction

Human locomotion requires the precise integration of feedforward and feedback neuromuscular control mechanisms to optimize stability, maneuverability, and metabolic efficiency (Ijspeert and Daley, 2023). When these neurological and biomechanical pathways are disrupted by lower-limb amputation, stroke, or progressive neurodegenerative conditions such as Parkinson’s disease, individual mobility and functional independence are severely degraded (Olson et al., 2019; Mollà-Casanova et al., 2022; Kesar, 2023). Consequently, affected individuals often exhibit pathological gait characterized by increased metabolic energy expenditure, distinct spatiotemporal asymmetries, and an increased risk of falls (Tucker et al., 2015; Manz et al., 2022). Conventional passive prostheses and orthoses provide essential structural support; however, their invariant mechanical properties limit their efficacy across varied terrains, ambulation speeds, and dynamic environments (Pickle et al., 2017; Kent et al., 2019). Consequently, users must execute significant compensatory movements, which often lead to chronic secondary musculoskeletal complications (Welke et al., 2019; Finco et al., 2022).

To mitigate these limitations, the field of wearable robotics has increasingly transitioned toward active, powered systems capable of delivering net positive joint work (Gehlhar et al., 2023). Despite substantial hardware advancements, traditional rule-based or deterministic control frameworks often exhibit diminished performance when encountering the high intra- and inter-subject variability characteristics of pathological locomotion. Current research is therefore focused on developing smart, co-adaptive human-machine interfaces. This paradigm shift relies on advancements in hybrid actuation, real-time sensor fusion, and adaptive machine learning algorithms designed to decode user intent, optimize system energetics, and dynamically modulate mechanical torque profiles (Chen et al., 2026).

### 1.1 Scope of the Research Topic

This Research Topic, titled ‘Advances in Smart and Adaptive Prosthetic and Wearable Technologies’, compiles five contributions that systematically address these scientific challenges across the actuation, perception, and control hierarchy. The primary objective of this Research Topic is to investigate and promote advanced methodologies that drive lower-limb prosthetics and wearable systems to become smarter, more adaptive, and deeply attuned to individual user needs.

## 2 Contributions

### 2.1 Hybrid actuation and biomechanics

Replicating the dynamics of biological gait requires hardware configurations capable of modulating power delivery based on real-time environmental and velocity contexts. To establish a rigorous baseline for these emerging technologies, Wang et al. present a systematic review investigating the intersection of actuation, perception, and control in intelligent prosthetic hips and knees. The authors chart a crucial technological migration away from isolated active or passive components toward hybrid active-passive architectures engineered to replicate the biarticular muscle-driven energy transfer of biological gait. By isolating persistent bottlenecks, such as sensory signal cross-talk, environmental classification delays, and the mathematical latency of real-time intention recognition algorithms, this review establishes a foundational framework that contextualizes the contributions within this Research Topic.

Directly addressing actuation dynamics at the joint level, Marsh et al. investigate the biomechanical implications of introducing powered swing extension assistance to a nominally passive, semi-powered prosthetic knee during fast walking speeds. Mirroring the active neuromuscular mechanisms utilized by healthy individuals at high velocities to accelerate swing extension, their active intervention yields a substantial increase in knee swing angular velocity. This response indicates that users prioritize mechanical and cognitive assurance that the knee has achieved full extension prior to weight-bearing, highlighting a critical human-in-the-loop adaptation. This study also reveals an essential tuning trade-off: elevated terminal impact at lower velocities causes user dissatisfaction, highlighting the necessity for highly adaptive, velocity-dependent control laws.

### 2.2 Machine learning and optimization

Transitioning to real-world ambulation requires replacing rigid control laws with data-driven modeling and machine learning architectures capable of personalizing device behavior. Scherpereel et al. successfully deploy machine-learning-based gait-phase estimation models within a wearable hip exoskeleton designed to navigate the extreme gait variabilities of Parkinson’s disease. Utilizing a synchronized flexion and extension torque profile driven by these adaptive algorithms, the device achieves clinically significant improvements, including an increase in both hip range-of-motion and peak toe clearance. The authors demonstrate that participant-specific models reduce gait-phase estimation errors by 40%, proving that data-driven personalization is key to maximizing control fidelity in highly variable clinical populations. While Scherpereel et al. leverage machine learning for real-time kinematic control, Schneider et al. utilize predictive analytics to decipher the hidden metabolic costs of the human-machine interface. Translating robotic power into a net reduction in user effort is exceptionally difficult due to the multi-dimensional nature of human energetics. The authors confront this challenge by training a machine learning interaction model on a comprehensive 50-feature dataset of spatiotemporal, kinetic, and kinematic variables to map their effects on the metabolic rate of walking with a powered ankle-foot prosthesis. This work provides a rigorous mathematical blueprint for personalized parameter tuning or targets for user training with a powered prosthesis. By ranking the contribution of individual biomechanical variables and how they should be changed to reduce metabolic rate, the authors identify the frontal plane kinematic features and step-to-step transition features among the most important biomechanical drivers of metabolic expenditure.

### 2.3 Reflexive control and fall mitigation

While steady-state gait optimization represents a significant milestone, a truly smart assistive device must also preserve user safety during locomotive hazards, where rule-based control systems frequently fail. King et al. evaluate the prospective efficacy of a powered knee prosthesis configured with bimodal stumble recovery behaviors modeled directly after able-bodied neuromuscular reflexes. Given that traditional mechanical prostheses leave individuals with transfemoral amputation vulnerable to increased fall risk after a trip or stumble, this reflex-driven intervention represents a major safety advancement. The active system successfully eliminates prosthetic knee buckling, enhances trunk control, and mitigates compensatory strategies like hip circumduction across varying stumble conditions. This work underscores how active torque generation, when coupled with rapid intent perception, can transform prostheses from passive mechanical devices into active aids capable of preserving user stability in real-time.

## 3 Conclusion and future horizons

The contributions compiled within this Research Topic demonstrate that the boundary of lower-limb wearable robotics is increasingly defined by the perception and control intelligence of the human-machine interface rather than raw mechanical capacity. By translating systematic review frameworks into targeted joint actuation (Wang et al.; Marsh et al.), applying biomimetic reflexes for active fall mitigation (King et al.), and utilizing machine learning to decipher both metabolic costs and pathological gait signatures (Schneider et al.; Scherpereel et al.), these works provide a cohesive vision for the future of assistive technologies.

These studies demonstrate that achieving true human-machine symbiosis requires transitioning from rigid control paradigms toward agile, personalized, and co-adaptive systems. Resolving the remaining translational bottlenecks will require ongoing, interdisciplinary integration across robotics, machine learning, and rehabilitation engineering. Ultimately, this Research Topic establishes a foundational benchmark for the next-generation of wearable devices that seamlessly learn, evolve, and adapt to their users, transforming prosthetics and exoskeletons into intuitive extensions of the human body that restore safety, autonomy, and quality of life.
